# Pleiotropic Effects of IGF1 on the Oocyte

**DOI:** 10.3390/cells11101610

**Published:** 2022-05-11

**Authors:** Paweł Kordowitzki, Kornelia Krajnik, Agnieszka Skowronska, Mariusz T. Skowronski

**Affiliations:** 1 Department of Immunology and Pathology of Reproduction, Institute of Animal Reproduction and Food Research of Polish Academy of Sciences, 10-243 Olsztyn, Poland; 2 Department of Basic and Preclinical Sciences, Faculty of Biological and Veterinary Sciences, Nicolaus Copernicus University, 87-100 Torun, Poland; skowron@umk.pl; 3 Department of Animal Physiology and Neurobiology, Faculty of Biological and Veterinary Sciences, Nicolaus Copernicus University, 87-100 Torun, Poland; kornelia.krajnik@umk.pl; 4 Department of Human Physiology and Pathophysiology, School of Medicine, Collegium Medicum, University of Warmia and Mazury, 10-719 Olsztyn, Poland; agnieszka.skowronska@uwm.edu.pl

**Keywords:** IGF1, insulin like growth factor, oocyte, aging, IVF, liver, ovary

## Abstract

A woman’s endocrine system plays a crucial role in orchestrating cellular interactions throughout her life. The growth hormone (GH) and insulin-like growth factor (IGF) system appears to impact crucial reproductive events and cell types of the ovary, such as granulosa cells, theca cells, and oocytes. Further, IGF1 is a cornerstone during embryonic development and influences predominantly developing and pre-antral follicles. In this commentary, we will emphasize the pleiotropic effects of IGF1 on physiological processes inside the egg. Herein, we will provide a brief overview on IGF1 related cell signal transduction pathways during the maturation and aging of oocytes. We aim to elucidate from a molecular and biochemical point of view if IGF1 in women with metabolic imbalances such as obesity or diabetes could be used in clinics as a novel, reliable estimator for the developmental competence of an oocyte.

## 1. Introduction

Though fundamental advances have been generated in the field of reproductive medicine and assisted reproductive technologies, molecular factors and pathways which could be pivotal for the aging process of oocytes remain elusive [1]. Hence, further research and novel therapeutic strategies related to the regulation of oocyte maturation and aging are required. The burden of reproductive senescence in women on public health is of high relevance, especially nowadays [2]. This is compounded by increased mean life expectancy that is not matched by women’s reproductive lifespan. Since the 1960s, in order to pursue higher education and reach certain career goals in modern demanding society, women and couples are postponing their first pregnancy [3]. Data from the last three decades has shown that fertility of women reaches a peak at approximately 25 years of age, and past the age of 35 years women experience a precipitous decline of their fertility [4,5]. As a consequence, a growing number of women depends on Assisted Reproductive Technologies (ART) to get pregnant. In 2019, prior to the COVID-19 pandemic, the Society for Assisted Reproductive Technology (SART) reported in total 298,689 cycles of oocyte retrieval in all SART member clinics in the USA [6]. Conversely, the aging phenotype of oocytes is a complex puzzle due to women’s lifestyles and diets, environmental events, their genetic background, oxidative stress, and epigenetic alterations accumulated from birth to menopause [7].

## 2. The Influence of Insulin-like Growth Factor-1 on the Oocyte

**Figure 1:**
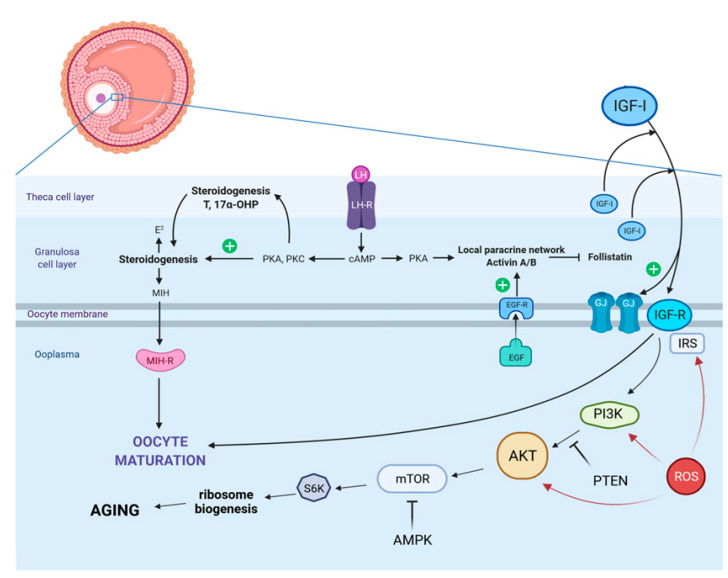
This scheme shows the influence of IGF1 on the maturation and aging process of oocytes. In vivo, the maturation of oocytes is stimulated by the pre-ovulatory surge of the luteinizing hormone (LH). The LH binds to LH receptors on mural granulosa cells and generates the production of specific epidermal growth factor-like peptides that transmit signals to the cumulus cells and the oocyte itself. The gonadotropins are commonly combined with epidermal growth factor (EGF) or EGF-like factors that are produced by follicular cells. It has been convincingly demonstrated that insulin-like growth factor 1 (IGF1) enables the maturation of oocytes and aging through a phosphoinositide-3-kinase/v-akt murine thymoma viral oncogene homolog (PI3K/AKT)-dependent mechanism. Abbreviations: AMPK: activated protein kinase; GJ: gap-junctions; IGFR: insulin-like growth factor receptor; IRS: insulin receptor substrate; MIH: maturation-inducing hormone; MIH-R: maturation-inducing hormone receptor; mTOR: mammalian target of rapamycin, PTEN: Phosphatase and Tension Homolog; ROS: reactive oxygen species; S6K: S6 kinase; green plus: positive/activating effect; red arrows: negative effect.

The IGF family consists of IGF1 and IGF2 polypeptide ligands, their respective receptors, IGFR1 and IGFR2, and also six binding proteins (IGFBP1–6) [8]. Interestingly, once IGF1 is released it acts through a paracrine/autocrine mode on granulosa cells and on the oocyte, regulating cell proliferation, differentiation, survival, and steroidogenesis as well as oocyte maturation [9]. As confirmed in a recent study, the administration of IGF1 at a concentration of 100ng/mL stimulated primordial follicle activation, and led to a reduction of DNA fragmentation through the PI3K/AKT pathway (Figure 1) [10].

The presence of IGF1 has been detected in every oocyte developmental stage. This may suggest that this factor is directly involved in the development and maturation of these cells. It has also been indicated that such involvement may not remain indifferent to the quality of oocytes. Studies have shown that overstimulation with IGF1 may lead to the deterioration of the quality of oocytes, which strengthens this theory. Based on this, it was concluded that IGF1 may interfere with the embryo’s developmental process and affect its chances of survival [11,12,13]. The latter mentioned fact, namely that IGF1 seems to improve oocyte quality and in consequence to this improves early embryonic development and blastocyst formation, was confirmed by numerous studies in different species [14,15,16,17].

Despite the fact that the molecular basis of aging is limited to few highly evolutionarily conserved biological pathways, a woman’s endocrine system plays a crucial role in orchestrating cellular interactions during oocyte maturation and aging [18]. With regards to this, the pleiotropic effects of the insulin-like growth factor 1 (IGF1)/insulin system gained researchers’ and doctors’ attention when discussing how to slow down oocyte aging or how to extend oocyte’s longevity. More precisely, IGF1 is an important cornerstone during physiological embryonic development and tissue growth [19]. Noteworthy, the liver is the major organ producing IGF1 might have auto-, para-, and/or endocrine effects [20], and its signal is transduced through the insulin-like growth factor 1 receptor (IGF1R) (Figure 1) [21]. Alterations of this pathway due to maternal malnutrition, obesity, or endocrinology imbalances impact the female embryo very early in life, and could have tremendous intrauterine effects. IGF1 might influence the primordial germ cells of a female embryo, before forming oogonia. A previous study provided strong evidence that circulating IGF1 was reduced in rodent neonates which experienced intra-uterine growth restriction (IUGR), while the protein abundance of IGF1 in the liver was significantly increased [22]. Therefore, it is of high importance for women who are planning to get pregnant or who are pregnant already to pay special attention to their nutrition and metabolism. Maternal obesity was shown to provoke IUGR, consequently impacting the resulting offspring [23]. In the short reproductive lifespan of a female individual, the fate of the earlier mentioned oogonia is multifaceted; very few of them develop as oocytes, and are selected to be ovulated. Previous studies provided strong evidence that the crosstalk between the insulin receptor substrate (IRS) and the modulation of the PI3K/AKT mammalian target of rapamycin (mTOR) pathway (Figure 1) impacts the pathogenesis of several aging-related diseases [24,25]. Interestingly, it was revealed that centenarians have a higher insulin sensitivity and a more adequate preservation of beta-cell function than younger counterparts [18]. Functional mutations in the IGF1R which affects IGF1 signaling, are present at a higher rate in centenarians [18], and have been shown to be involved in intrauterine and postnatal growth retardation [26]. Surprisingly, diminished concentrations of IGF1 in women seem to be a general survival advantage [18]. Importantly though, these findings could be extrapolated to the oocyte since IGF1 might be a powerful hallmark for maternal aging.

Transcripts of IGF1R/IGF2R have been detected in unfertilized oocytes and IGF1R appears to be necessary for the differentiation of human cumulus granulosa cells. The cumulus cell’s response to FSH resembles the differentiation of preantral to preovulatory granulosa cells. This differentiation program requires the activity of IGF1R and subsequent AKT activation [27] (Figure 1). Data generated from bovine granulosa cells revealed that the IGF1R increases FSH and LH receptors [28,29]. When using quantitative PCR, a significant signal for *IGF1R* mRNA was measured in germinal vesicle (GV) stage oocytes. Noteworthy, in a mutant mice model it has been shown that female reproductive functions, oocyte development and maturation, and litter size are not diminished when INSR or IGFR1 was ablated in oocytes [30]. Moreover, it was suggested that the IGF1R mainly facilitates IGF1 action in granulosa cells. Therefore, the lack of IGF1R signaling in the latter mentioned cells is correlated with an elevated apoptosis level at all stages of follicular development [31].

## 3. Clinical Aspects of Testing for Insulin-like Growth Factor-1 in Sub-Fertile Women

With regards to the improvement of assisted reproductive technologies and in vitro fertilization stimulation protocols in the last three decades, growth hormone (GH) administration was introduced in the late 1980s. However, the effectiveness of the latter mentioned protocol with the aim to improve IVF outcomes remains controversial [32]. Many studies in the past have focused on the consequence of metabolic disorders, such as obesity and diabetes mellitus [33,34,35,36], on oocyte quality. There are only few articles published in which the composition of blood serum and follicular fluid was compared [33]. If a reliable correlation between these two latter mentioned fluids could be determined, a new diagnostic marker could be introduced in IVF clinics. In particular, the concentration of IGF1 would be of high interest with regards to maternal age or maternal Body Mass Index [33,34]. A previous study provided evidence that most changes in serum IGF1 levels are mirrored in the follicular fluid [33]. Noteworthy, peripheral IGF1 concentrations in sub- or infertile women have been investigated only in few reports [32,37]. Interestingly, it has been shown in a case report about a patient suffering from hypopituitarism that elevation of GH/IGF1 in the follicular fluid during a GH replacement therapy was linked with the normalization of abnormal oocytes, and the embryonic quality and outcome was improved when undergoing IVF procedure [38,39].

As confirmed in a recent study, the administration of IGF1 at a concentration of 100ng/mL stimulated primordial follicle activation, and led to a reduction of DNA fragmentation through the PI3K/AKT pathway (Figure 1) [10]. There is also no clear consensus about the desirable concentration of IGF1 in blood serum or follicular fluid. On the one hand, some authors defined high IGF1 concentrations when reaching the threshold of about 72.0 ng/mL [40], while in the study of Gleicher et al. this border was defined at IGF1 level of 132 ng/L. Further, Gleicher et al. hypothesized that GH administration seems to be indicated in patients with low peripheral IGF1 levels [32]. Interestingly, a study on bovine follicle development revealed that the effects of IGFI are dose and stage dependent [41].

So far there is limited data available about the peripheral IGF1 concentrations in infertile women [32]. Additionally, the supplementation with Growth Hormone (GH) to stimulate the IGF1 pathway remains controversial. Several translational animal models were used in the past to elucidate the relevance of IGF1 for reproductive aging. In a murine model, knockout of the IGF1 gene led to infertility and dwarfism, underlying the influence of IGF1 already during the embryonic phase [32]. In a recent study, the effects of follicular GH and IGF1 levels on oocytes which undergo IVF have been investigated [32]. The authors of the latter-mentioned study postulate that GH supplementation should only be considered for women with IGF1 levels below the physiological range. Further, it was hypothesized that the pleiotropic effects of IGF1 occur predominantly in developing, pre-antral follicles [32]. These follicles are bearing the oocyte which is recognized as the largest cell in mammalian species and other multicellular organisms [32]. Interestingly, IGF1 appears to have also an effect on mitochondria bioenergetics and metabolism [42]. Even though the rate of mitochondria in oocytes is higher in comparison to other mammalian cells, their number and activity decrease with increasing maternal age [1]. Consequently, the crosstalk between IGF1 and mitochondria is of high relevance for the human egg.

That being said, mitochondria are not only the central powerhouse [40], but also a crucial cornerstone for the production of several precursor molecules that form blocks for protein, lipid, DNA, and RNA biosynthesis, and mediate metabolic waste products [1,43]. Mitochondria are well-known as redox signaling hubs, and opposed to the damaging roles of ROS; they also have pleiotropic effects as signaling molecules [1] (Figure 1). It is worth mentioning that advanced maternal age and female fertility is negatively correlated in different mammalian species. With regards to evolution, it appears to be more desirable for a mother to give birth to her offspring at younger age to ensure adequate maternal energy for care and survival of the newborn. This maternal effect on senescence was described as reproductive senescence, meaning that there is a relevance for evolution since age-specific selective pressures to fertility occur. It has been described that fertility and maternal effect on senescence seem to show different patterns of age-specific selection [44]. Population genetic models provided evidence that maternal effects can advance in the absence of reproductive or actuarial senescence. In consequence, maternal aging has been reported to be a fundamentally distinct demographic manifestation of the evolution of aging [41].

## 4. Conclusions

In conclusion, there is no doubt that more studies on human patients are required to assess the adequate IGF1 concentrations both in blood serum, and follicular fluid for the best oocyte quality and IVF outcome. More studies involving larger patient numbers are needed to provide reliable answers to the question how the manipulation of the IGF1 system in IVF patients could be used in clinics with regards to a defined patient with a low success rate. It would be interesting to test if in younger women with low IGF1 levels there would be a positive effect upon the GH treatment. Moreover, the hypothesis that the before-mentioned supplementation seems to increase IVF success rate only in patients with low IGF1 concentrations has to be investigated in depth. All in all, due to the controversial discussion about GH supplementation and IGF1 levels, this Comment aimed to stimulate researchers to perform further studies on the pleiotropic effects of IGF1 on the oocyte, especially in women of advanced age and in those patients with GH/IGF1 deficiencies, including patients with diabetes mellitus or other metabolic disorders.
